# Letter migration errors reflect spatial pooling of orthographic information

**DOI:** 10.3758/s13414-019-01746-z

**Published:** 2019-05-06

**Authors:** Aaron Vandendaele, Joshua Snell, Jonathan Grainger

**Affiliations:** 1 Department of Experimental Psychology, Ghent University, Ghent, Belgium; 2 Laboratoire de Psychologie Cognitive, CNRS & Aix-Marseille University, 3 place Victor Hugo, 13331 Marseille, France; 3 Institute for Language, Communication and the Brain, Aix-Marseille University, Marseille, France

**Keywords:** Orthographic processing, Letter migrations, Parallel processing, Flanker paradigm, Letter positions

## Abstract

Prior research has shown that readers may misread words by switching letters across words (e.g., the word *sand* in *sand lane* being recognized as *land*). These so-called letter migration errors have been observed using a divided attention paradigm whereby two words are briefly presented simultaneously, and one is postcued for identification. Letter migrations might therefore be due to a task-induced division of attention across the two words. Here, we show that a similar rate of migration errors is obtained in a flanker paradigm in which a central target word is flanked to the left and to the right by task-irrelevant flanking words. Three words were simultaneously presented for the same brief duration. Asked to type the target word postoffset, participants produced more migration errors when the migrating letter occupied the same position in the flanker and target words, with significantly fewer migrations occurring across adjacent positions, and the effect disappearing across nonadjacent positions. Our results provide further support for the hypothesis that orthographic information spanning multiple words is processed in parallel and spatially integrated (pooled) within a single channel. It is the spatial pooling of sublexical orthographic information that is thought to drive letter migration errors.

## Introduction

Much of psycholinguistic research has focused on how linguistic processes can operate perfectly, but it may be equally interesting to look at how processing can go awry. One area where this has been particularly informative is that of language production, with well-known examples of speech errors such as spoonerisms and slips of the tongue providing insights with respect to the basic mechanisms involved in producing speech (e.g., Dell & Reich, 1981). In a similar vein, looking at errors that are based on written language input might provide insight with respect to orthographic processing and, in particular, with respect to one question that has attracted much attention in recent years: the representation and encoding of letter position information (see Grainger, 2008). The present study examines one specific type of error that is related to the representation of letter position information: letter migration errors.

Previously, the paradigm of choice to elicit letter migration errors was a divided attention paradigm (Allport, 1977; Davis & Bowers, 2004; McClelland & Mozer, 1986; Mozer, 1983; Shallice & McGill, 1978). In these studies, participants were briefly presented with two different words, followed by postmasking of those words. After that, one of the words was cued for verbal report (or a single letter cued in certain experiments). A consistent finding is that letter migrations occur to form illusory words—that is, words that were not shown to participants. For example, upon presentation of the words SAND and LANE and cued to report the word on the left, participants would incorrectly report LAND instead of SAND, and significantly more so than when the word on the right was a control word such as BANK (Mozer, 1983). One key finding is that letter migrations are more likely to occur between orthographically similar words than between words that have no letters in common, referred to as the surround-similarity effect (McClelland & Mozer, 1986). Another key finding is that the migrating letter does not have to remain at the same position (e.g., a letter on Position 2 in the noncued word could migrate to Position 3 in the cued word; Davis & Bowers, 2004). These two findings point to a system that (1) is able to integrate orthographic information across different words into a single processing channel (McClelland & Mozer, 1986), and (2) allows for a certain level of uncertainty or flexibility in letter position coding (Davis & Bowers, 2004).

However, this evidence for the spatial pooling of orthographic information from different words was obtained in conditions that encourage participants to divide their attention across the two words. One could argue that this is very different from more natural reading behavior where attention is thought to be largely focused on one word at a time (e.g., Reichle, Pollatsek, Fisher, & Rayner, 1998). However, even in sentence reading, it has been found that word-recognition speed is influenced by the extent to which words are orthographically related to upcoming words (e.g., Angele, Tran, & Rayner, 2013; Dare & Shillcock, 2013; Snell, Vitu, & Grainger, 2017), which suggests either that the spatial pooling of orthographic information proceeds preattentively (e.g., Angele et al., 2013), or that attention is inevitably directed to multiple words at once.

In line with the latter findings, evidence for the spatial pooling of orthographic information spanning multiple stimuli without encouraging divided attention has been obtained using the flanking letters lexical decision task (Dare & Shillcock, 2013). Here, participants have to perform a lexical decision task on centrally presented words flanked by letters located to the left and to the right of targets and separated from the target by a space. In Dare and Shillcock’s (2013) seminal study, the flanking letters were bigrams formed from the first two and last two letters of targets in the related condition (e.g., ro rock ck), and different letters in the unrelated condition (e.g., pa rock th). The important finding here is that not only did related flankers facilitate lexical decisions to target words when the bigrams respected their order in the target but they did so to the same extent when bigram order was switched (e.g., ck rock ro). This finding was replicated by Grainger, Mathôt, and Vitu (2014), who further demonstrated that reversing the order of letters in bigrams (e.g., or rock kc) caused a significant reduction in priming.^1^ Grainger et al. (2014) interpreted these findings within the framework for orthographic processing proposed by Grainger and van Heuven (2004). According to this account, orthographic information from both parafoveal flanker and foveal target stimuli are spatially integrated (pooled) into a single processing channel. Flanking letters can then contribute to the process of target-word identification by either providing a boost in activation to the target word’s component letters (in the case of related flankers, thus leading to facilitation) or providing negative evidence for the target word in the case of unrelated flankers, hence leading to inhibition. Here, it is important to note that Grainger et al.’s (2014) account of spatial pooling of orthographic information based on evidence from the flanker paradigm embodies the two main conclusions derived from studies of letter migration errors using the divided attention paradigm: namely, that orthographic information is pooled beyond single words, and that letter position coding is subject to a certain amount of flexibility.

Given that results obtained with the flanker paradigm reflect spatial pooling of sublexical orthographic information (i.e., letters and/or letter combinations), and that the flanker paradigm and the divided attention paradigm seem to provide different windows on the same process, we therefore predicted that we should be able to observe letter migration errors in the flanker paradigm. The current study was designed to test this prediction by using stimuli mimicking those tested in one prior letter migration study (Davis & Bowers, 2004), and adapting the flanker paradigm to the brief presentation and masking conditions used in prior letter migration experiments. Finding letter migration errors in the flanker paradigm would provide valuable evidence that prior reports of letter migration errors are not due to any specificities of the paradigm that was used, and in particular that such errors did not occur because participants were encouraged to divide their attention across multiple words. The use of target-word identification rather than lexical decision as a task would further yield a more direct view on how the word-recognition process is influenced by surrounding information. Whereas lexical decisions in our previous flanker experiments arguably gave insight with respect to how flankers influenced the experienced “wordlikeness” of target stimuli, a target identification task will tell us concretely whether the spatial pooling of orthographic information can indeed lead to erroneous recognition.

In the present study, participants had to identify a single, centrally located target word that was flanked by the same flanking word to the left and to the right. We manipulated the orthographic overlap across target and flankers in order to induce migration errors. For example, if the following target and flankers, *folie farce folie*, are presented, the letter *o* in *folie* can migrate to replace the letter *a* in *farce* thus inducing the migration error *force*. Following Davis and Bowers (2004), we also investigated migrations to different letter positions in target and flankers.

## Method

### Participants

Fifty-six students (47 female) from Aix-Marseille University gave written consent to partake in this experiment and received monetary compensation (at the rate of €10/hour) or course credit. All participants were native French speakers, reported to have normal or corrected-to-normal vision, and ranged in age from 18 to 32 years (*M* = 22.1, *SD* = 3.5).

### Stimuli and design

We selected 180 five-letter target–flanker word pairs from the French Lexicon Project database (Ferrand et al., 2010). The targets had a mean frequency of 3.86 ZipF (van Heuven, Mandera, Keuleers, & Brysbaert, 2014) and were specifically chosen such that if a critical letter was replaced with a different letter drawn from the corresponding flanker word (referred to as the *migration flanker*), a new word could be formed, referred to as the *illusory word* (e.g., the replacing the second letter in the target word *farce* with the second letter from the flanker word *folie* produces the illusory word *force*). Illusory words were therefore orthographic neighbors of the target words. Control flanker words were chosen so that no possible combination of letter migrations could result in an existing five-letter French word. All target, migration flanker, and control flanker word triplets had the same initial and final letters, with migrations only possible at Positions 2, 3, or 4 (see Table 1). The target words were from the following grammatical categories: nouns (64%), verbs (29%), and adjectives or prepositions (7%). There were no diacritics (e.g., *á, è, ï, û, ç*) in either the target, flanker, or illusory words. On each trial, the target word was flanked to the left and to the right by either the corresponding migration flanker word or by the matched control word. A small percentage of words could appear twice (e.g., a word that was a target in one trial could be a flanker or an illusory word in another), but no word appeared as a target more than once per condition. We further manipulated the distance (in number of letters) separating the position of the migrating letter in the migration flanker word and in the illusory word. This distance could either be 0 (same position), 1 (adjacent position), or 2 (distant position; see Table 1 for examples). This resulted in a 2 (migration flanker vs. control flanker) × 3 (distance) factorial design. The average target word/migration flanker word/illusory word frequencies (Zipf)^2^ in the three distance conditions were 3.86/4.10/4.12; 4.00/3.78/3.99; 3.72/3.63/3.87. All target words were seen twice by all participants—once with the migration flankers and once with the control flankers. Each set of target words was split in two, and two stimulus lists were created such that in each list half of the targets were paired with a migration flanker and the other half with a control flanker using a Latin-square design. The presentation order of the two lists was counterbalanced across participants. The experiment thus consisted of 360 trials, presented to participants in random order.

**Table 1:** Examples of stimuli tested in the experiment

Distance	From → To position	Target word	Migration flanker	Control flanker	Illusory word
0 (same)	2 → 2	NUIRE	NOBLE	NETTE	NOIRE
	4 → 4	COULE	CAMPE	CACHE	COUPE
1 (adjacent)	2 → 3	HALTE	HUILE	HORDE	HAUTE
	4 → 3	TIGRE	TANTE	TOQUE	TITRE
2 (distant)	2 → 4	PLATS	PNEUS	PEURS	PLANS
	4 → 2	AGILE	ABUSE	ADORE	ASILE

*Note.* Distance refers to the number of letter positions (0, 1, 2) separating the position of the migrating letter in the flanker and the illusory word. Position (2, 3, 4) refers to the position in the flanker (from) and position in the illusory word (to). The migrating letter and the corresponding letter in the target is underlined in these examples for illustration purposes

### Apparatus

The stimuli and experimental design were implemented with OpenSesame (Mathôt, Schreij, & Theeuwes, 2012) and presented on a 24-inch 1,024 × 768-pixel LCD-screen. Participants were seated at an 80-cm distance from the display, so that each character space subtended 0.24° of visual angle. All words were presented in lowercase using a 24-point monospaced font (droid sans mono, the standard in OpenSesame). All responses were collected via a computer keyboard.

### Procedure

**Fig. 1:**
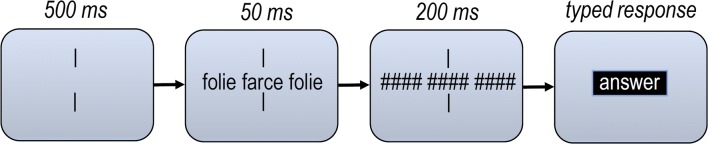
Procedure of the flanker task used in the present study. Participants had to type in the identity of the central target word

Participants were seated in a comfortable office chair in a dimly lit room. Before the experiment, instructions were given both verbally and visually on-screen. Every trial began with vertically aligned fixation bars that stayed on-screen for 500 ms. After that, the target word and flankers appeared for 50 ms (each word separated by a single character space). Both target and flanker words were then replaced by masks that consisted of five hash marks (‘#’), which stayed on-screen for 200 ms. After that, a target box appeared one line below the central word in which participants could type their response. Responses could be corrected if necessary using the backspace key. After registering their responses using the return key, a new trial would start (see Fig. 1 for a summary of the procedure). Before the main experiment, 10 practice trials were presented that gave feedback in the form of a green (correct) or red (incorrect) dot. No feedback was given during the main experiment. Participants were offered a break at the halfway point. The experiment lasted approximately 25 minutes.

## Results

The overall average error rate was 23.37% (*SD* = 8.46%). Incorrect responses were categorized either as a letter migration, a word migration, a neighbor migration, or other error. Letter migration errors constitute the report of an illusory word (i.e., a word formed by replacing a letter in the target word with a letter from the migration flanker word, such as reporting SHARE instead of the target SHAME when the flanker is SCARE).^3^ A word migration error occurred when the flanker word was reported instead of the target, and a neighbor migration error occurred when an existing word was reported that is an orthographic neighbor of the flanker word (e.g., reporting SCORE when the target is SHAME and the flanker is SCARE). The remaining errors were classified as “other” and consisted of four-letter words, pseudowords, spelling errors (i.e., five-letter words orthographically similar to the target word), blank responses, or completely different five-letter words. A detailed break-down of these percentages is shown in Table 2.

**Table 2:** Average percentages of response types per condition

Condition		
Response type	Migration	Control
Correct	75.87	77.29
Letter migration	3.57	1.69
Word migration	2.78	3.23
Neighbor migration	0.08	0.17
Other	17.68	17.62

We used generalized linear mixed models to analyze differences in error rates across conditions, with participants and items as crossed random effects (Baayen, Davidson, & Bates, 2008; Barr, Levy, Scheepers, & Tily, 2013). This was done using the glmer function from the lme4 package (Bates, Mächler, Bolker, & Walker, 2015). We report regression coefficients (*b*), standard errors (*SE*) and *z* values. Fixed effects were deemed reliable if |*z*| > 1.96 (Baayen, 2008). All analyses were done with the Rstudio (Version 3.4.2) statistical computing environment. We focus on letter migration errors and only report other effects when significant.

### Letter migration errors

We observed a total of 361 word reports out of all the trials from the letter migration condition (which equals 3.57%). At first sight, this number might look rather small, but bear in mind that the majority of trials were answered correctly (75.87%). To examine whether our manipulation was successful at inducing letter migrations we looked at the number of times an illusory word was reported as a result of our manipulation (i.e., with a migration flanker) versus when it was reported as an error that could not have resulted from a migration (the control condition). We also examined the impact of the distance between the position the migratory letter occupied in the flanker versus the position it occupied in the illusory word (same, adjacent, distant: see Table 1 for examples, and Table 3 for results). We found a significant main effect of condition (*b* = −0.86, *SE* = 0.10, *z* = −8.68), meaning that an illusory word was more likely to be reported in the migration condition.^4^ We also observed a main effect of distance (*b* = −1.99, *SE* = 0.39, *z* = −5.12). Crucially, there was a significant interaction between condition and distance (*b* = −1.95, *SE* = 0.25, *z* = −7.63).^5^ As can be seen in Table 3, the interaction reflects the monotonic decrease in the size of letter migration effects (migration − control) as a function of distance. It also reflects the fact that the effect of distance was significant in the migration condition (*b* = −2.38, *SE* = 0.49, *z* = −4.87), but not in the control condition (*b* = −0.62, *SE* = 0.48, *z* = −1.29).

**Table 3:** Letter migration effects as a function of migration distance

Migration distance	Migration	Control	Difference	*b*	*SE*	*z*
0 (same)	6.90	1.64	5.26	−1.67	0.16	**−10.42**
1 (adjacent)	2.14	1.39	0.75	−0.44	0.19	**−2.22**
2 (distant)	1.64	1.99	−0.35	0.23	0.19	1.18

*Note*. Data are the average percentage illusory word reports with letter migration flankers (Migration) and control flankers (Control). Migration distance (0, 1, 2) represents the number of letter positions separating the position of the migrating letter in the flanker and its position in the illusory word. Significant values are shown in bold

### Word migration errors

Comparing the number of flanker words reported instead of target words in the letter migration and control conditions revealed a significant effect of condition (*b* = 0.18, *SE* = 0.08, *z* = 2.11), with more flanker words being reported in the control condition.

### Frequency effects

In a final analysis, we examined the effects of word frequency on the number of illusory word reports due to letter migrations. The frequency values (Zipf) for the target words, the illusory words, the migration flanker words, and the control flanker words were entered as continuous variables along with the condition variable. The results for the target and illusory words are shown in Table 5. Flanker word frequency had no significant influence (migration flanker: *b* = -0.15, *SE* = 0.23, *z* = −0.65; control flanker: *b* = −0.29, *SE* = 0.20, *z* = −1.47). As can be seen in Table 4, there was no main effect of target-word frequency, but a significant interaction with condition. Target-word frequency only affected illusory word reports in the control condition. There was a main effect of illusory word frequency and an interaction with condition. Illusory word frequency had a significant impact on illusory word reports in both conditions, but the effect was greater in the control condition.

**Table 4:** Effects of target word frequency and illusory word frequency on illusory word reports

	Target word			Illusory word		
	*b*	*SE*	*z*	*b*	*SE*	*z*
Frequency (F)	−0.24	0.17	−1.43	1.19	0.19	**6.19**
Condition x F	−0.48	0.11	**−4.39**	−0.37	0.16	**−2.28**
F/Migration	−0.12	0.26	−0.51	0.79	0.24	**3.29**
F/Control	−0.90	0.20	**−4.45**	0.86	0.23	**3.75**

*Note*. The two bottom lines show the effects of target word and illusory word frequency separately for the migration and control conditions. Effects of flanker word frequency were not significant (*z* < 1.5). Significant values are shown in bold

## Discussion

The main goal of the present experiment was to examine whether letter migration errors can be observed in a flanker paradigm in which target words are centrally located and where focusing all attentional resources solely on the target would be beneficial for the task. The aim was to demonstrate that prior observations of letter migration errors using a divided attention paradigm (e.g., Davis & Bowers, 2004; McClelland & Mozer, 1986; Mozer, 1983) were not the result of processes triggered by the fact that participants were encouraged to identify two words at the same time in these studies. Our results suggest, indeed, that this was not the case, because we were successful in inducing letter migration errors in the flanker paradigm where only one word has to be identified. Here, we will argue that it is the spatial pooling of sublexical orthographic information that is the cause of letter migration errors seen in both the divided attention paradigm and the flanker paradigm.

An important finding of the present experiment is that we observed a level of letter migration errors that is comparable with that found in the third experiment of Davis and Bowers (2004), upon which the present experiment was based. Although Davis and Bowers (2004) observed a greater percentage of letter migration errors overall, the relative error rate (relative to the total amount of errors) was comparable in their Experiment 3 and our experiment (18.83% vs. 14.85%). This simply suggests that it is harder to identify target words in the divided attention paradigm than in the flanker paradigm, leading to more errors overall, but that a similar mechanism is driving letter migration errors in the two paradigms, thus leading to a similar percentage of this type of error.

The second important finding of the present study concerns differences in the size of the letter migration effect as a function of the distance (in number of letters) between the position of the migrating letter in the flanker and the illusory words (0, 1, or 2). We found that the letter migration effect significantly diminished as the distance increased, to the point that it disappeared with the distant (two letter position difference) migrations (see Table 3). Davis and Bowers (2004) reported a similar monotonic decrease in the letter migration effect as a function of distance, but nevertheless found a significant effect of letter migrations with distant migrations. The fact that we failed to find an effect with distant migrations is likely due to the overall lower number of letter migrations obtained in the flanker paradigm. The key finding, nevertheless, is the significant interaction between the letter migration effect and migration distance. This finding fits with most current models of letter position coding (Davis, 2010; Dehaene, Cohen, Sigman, & Vinckier, 2005; Gomez, Ratcliff, & Perea, 2008; Grainger & van Heuven, 2004; Whitney, 2001), except for an unconstrained open-bigram model. Within the framework of open-bigram coding, used by Grainger et al. (2014) to account for spatial pooling of orthographic information, this pattern of results suggests either that more weight should be assigned to contiguous bigrams than to noncontiguous ones, or that an unconstrained open-bigram code must be complemented with a more precise position-coding mechanism using word edges (see Grainger, Dufau, & Ziegler, 2016; Snell, Bertrand, & Grainger, 2018).

A third important finding is that frequency plays a crucial role in the reporting of illusory words (see Table 4). Given that the illusory word is an orthographic neighbor of the target, the influence of illusory word frequency can be taken as evidence that the higher the frequency of an orthographic neighbor, the more strongly it will be activated upon presentation of the target word and hence be incorrectly reported instead of the target word. This is simply another demonstration of the impact of high-frequency orthographic neighbors on target-word processing in data-limited identification tasks (Carreiras, Perea, & Grainger, 1997; Grainger & Jacobs, 1996; Grainger & Segui, 1990). It is important to note, however, that there is abundant evidence that the interfering effects of orthographic neighbors are also obtained in speed of responding in response-limited paradigms (e.g., Carreiras et al., 1997; Grainger, 1990) as well as with eye-movement recordings in a simplified reading task (Grainger, O’Regan, Jacobs, & Segui, 1989) and during sentence reading (e.g., Perea & Pollatsek, 1998). This suggests that orthographic neighbors are influencing online processing of target words and not just processes that are implemented when word identification fails. The general idea is that the lexical representations of orthographic neighbors can be activated in parallel with the target-word representation and compete for identification (see Grainger & Jacobs, 1996, for further discussion). Although significant effects of illusory word frequency were found in both the migration and control flanker conditions, the effect was significantly stronger in the control condition. This suggests that the contribution of letter migrations to illusory word reports somewhat dampened the impact of illusory word frequency on such reports, and fits well with our interpretation of letter migration errors as reflecting online orthographic processing of target words.

Finally, we also found more word migrations in the control condition than in the letter migration condition. This is most likely due to the greater evidence for the illusory word, compared with the flanker word, in the letter migration condition. This is the very basis of the letter migration effect. In other words, illusory words are reported more than flanker words when these illusory words can be formed (i.e., in the letter migration condition), hence reducing the overall report of flanker words in that condition. Similarly, we found that target-word frequency only had an impact on accuracy in the control condition. This again could be due to the influence of illusory word frequency reducing the impact of target word frequency in the letter migration condition.

Is there evidence for spatial pooling of orthographic information in more natural reading situations? The answer is clearly yes. As noted in the Introduction, Dare and Shillcock (2013) not only demonstrated such effects in the flanker paradigm but also in a sentence-reading experiment with eye-movement recordings. This was done by manipulating the orthographic overlap between the currently fixated word (the target) and the letter string immediately to its right (the parafoveal stimulus). Once readers’ eyes left the critical target word, the parafoveal stimulus became the normal continuation of the sentence. Thus, for example, participants read the following word sequence: “The store had a coat coat that week,” and when their eyes left the first occurrence of “coat,” the second occurrence was changed to “sale,” and participants had the impression they had read the syntactically correct sentence “The store had a coat sale that week.” This repetition condition was compared with “The store had a coat milk that week,” with the word “milk” changing to “sale” as readers’ eyes left the word “coat.” Dare and Shillcock found that target word viewing times were significantly reduced when the parafoveal stimulus was a repetition of the target, and also when it was an orthographically similar pseudoword (e.g., “coat”–“caot”; see Angele et al., 2013; Snell et al., 2017, for further evidence obtained in sentence reading with eye movements and with words and pseudowords that are orthographic neighbors). Given this evidence, we suspect that letter migrations are part and parcel of the normal process of reading.

To conclude, the present finding that letter migration errors occur in the flanker paradigm lends support to two major conclusions drawn on the basis of findings obtained with the divided attention paradigm: (1) that sublexical orthographic information is processed in parallel across distinct stimuli and spatially integrated into a single processing channel (McClelland & Mozer, 1986), and (2) that this pooling process operates on an orthographic code in which letter identities are not strictly associated with specific positions in a word (Davis & Bowers, 2004).

## Appendix

**Table 5:** List of stimuli

Target word	Position (from → to)	Migration flanker	Control flanker	Illusory word
Fondu	2 → 2	Fessu	Fallu	Fendu
Ruses	2 → 2	Ronds	Rails	Roses
Cache	2 → 2	Coure	Curie	Coche
Sobre	2 → 2	Salue	Selle	Sabre
Nuire	2 → 2	Noble	Nette	Noire
Malle	2 → 2	Mitre	Malte	Mille
Fosse	2 → 2	Femme	Farde	Fesse
Plume	2 → 2	Parie	Pense	Paume
Pinte	2 → 2	Poule	Palpe	Ponte
Farce	2 → 2	Folie	Feule	Force
Bases	2 → 2	Biens	Bonus	Bises
Clope	2 → 2	Chute	Carte	Chope
Roche	2 → 2	Rifle	Rende	Riche
Songe	2 → 2	Situe	Selle	Singe
Vaste	2 → 2	Verre	Viole	Veste
Batte	2 → 2	Boire	Beure	Botte
Mises	2 → 2	Mucus	Morts	Muses
Foire	2 → 2	Faune	Fende	Faire
Laque	2 → 2	Lotie	Lippe	Loque
Pages	2 → 2	Pious	Ponts	Piges
Rires	2 → 2	Rangs	Ronds	Rares
Rites	2 → 2	Rangs	Ruons	Rates
Peste	2 → 2	Pille	Parle	Piste
Soins	2 → 2	Sales	Sures	Sains
Parle	2 → 2	Poste	Pelte	Poire
Vague	2 → 2	Voire	Veste	Vogue
Jaune	2 → 2	Jette	Jolie	Jeune
Brins	2 → 2	Bauds	Buses	Bains
Sport	2 → 2	Shift	Salut	Short
Cible	2 → 2	Carpe	Coche	Cable
Renie	4 → 4	Ruade	Racle	Rende
Taupe	4 → 4	Tuile	Trime	Taule
Sonde	4 → 4	Sarge	Sable	Songe
Guise	4 → 4	Garde	Gomme	Guide
Frite	4 → 4	Femme	Fauve	Frime
Parts	4 → 4	Plocs	Pieds	Parcs
Filme	4 → 4	Foule	Farde	Fille
Phare	4 → 4	Pulse	Pompe	Phase
Moine	4 → 4	Meute	Marge	Moite
Bribe	4 → 4	Blase	Balle	Brise
Marge	4 → 4	Momie	Menue	Marie
Stade	4 → 4	Songe	Serpe	Stage
Meure	4 → 4	Mixte	Magie	Meute
Trous	4 → 4	Tapis	Temps	Trois
Serve	4 → 4	Sobre	Saine	Serre
Coule	4 → 4	Campe	Cache	Coupe
Fonds	4 → 4	Faits	Fumes	Fonts
Halle	4 → 4	Honte	Heure	Halte
Pairs	4 → 4	Puons	Pouls	Pains
Exige	4 → 4	Enfle	Entre	Exile
Garce	4 → 4	Guide	Golfe	Garde
Douze	4 → 4	Dance	Demie	Douce
Larve	4 → 4	Longe	Loupe	Large
Barbe	4 → 4	Boire	Biffe	Barre
Bagne	4 → 4	Bique	Bille	Bague
Taule	4 → 4	Tempe	Terme	Taupe
Plans	4 → 4	Ports	Poses	Plats
Soupe	4 → 4	Sente	Sache	Soute
Poids	4 → 4	Pulls	Parts	Poils
Crise	4 → 4	Comme	Cafte	Crime
Prise	2 → 3	Poche	Peche	Prose
Merle	2 → 3	Munie	Mante	Meule
Coups	2 → 3	Cries	Cabas	Corps
Aille	2 → 3	Agace	Abuse	Aigle
Folle	2 → 3	Furie	Faire	Foule
Crame	2 → 3	Cible	Chose	Crime
Saule	2 → 3	Slice	Sobre	Salle
Sorte	2 → 3	Suive	Salve	Soute
Onlce	2 → 3	Ogive	Offre	Ongle
Grave	2 → 3	Gilde	Gosse	Grive
Ouvre	2 → 3	Otage	Opine	Outre
Payer	2 → 3	Prier	Polir	Parer
Faite	2 → 3	Fusse	Ferme	Faute
Halte	2 → 3	Huile	Horde	Haute
Tapie	2 → 3	Troue	Tombe	Tarie
Corps	2 → 3	Cubes	Chics	Coups
Ville	2 → 3	Votre	Vache	Viole
Toits	2 → 3	Trams	Temps	Torts
Fixer	2 → 3	Fluor	Futur	Filer
Pacte	2 → 3	Probe	Poile	Parte
Hurle	2 → 3	Hisse	Hache	Huile
Dinde	2 → 3	Dogue	Dague	Diode
Outre	2 → 3	Ovale	Oigne	Ouvre
Sages	2 → 3	Slows	Sinus	Sales
Fumes	2 → 3	Finis	Fards	Fuies
Coton	2 → 3	Clown	Caban	Colon
Plaie	2 → 3	Purge	Ponce	Pluie
Suave	2 → 3	Singe	Sobre	Suive
Marre	2 → 3	Mufle	Morse	Maure
Filme	2 → 3	Frite	Fasse	Firme
Capte	4 → 3	Cuire	Chime	Carte
Tigre	4 → 3	Tante	Toque	Titre
Borde	4 → 3	Bague	Basse	Boude
Pompe	4 → 3	Parue	Pense	Poupe
Pouce	4 → 3	Peine	Paire	Ponce
Liens	4 → 3	Labos	Lupus	Lions
Piges	4 → 3	Pouls	Ponds	Piles
Dette	4 → 3	Drone	Dance	Dente
Vides	4 → 3	Volts	Vapes	Vites
Verte	4 → 3	Visse	Voice	Veste
Chats	4 → 3	Cocus	Coins	Chuts
Texte	4 → 3	Turne	Table	Tente
Mette	4 → 3	Magne	Malle	Mente
Votes	4 → 3	Vrais	Vapes	Voies
Rames	4 → 3	Rhums	Ronds	Rares
Parme	4 → 3	Pique	Ponde	Paume
Beige	4 → 3	Boule	Bande	Belge
Verge	4 → 3	Vaine	Vomie	Venge
Verve	4 → 3	Vaque	Vanne	Veuve
Amant	4 → 3	Argot	Assit	Amont
Sable	4 → 3	Situe	Serpe	Saule
Salve	4 → 3	Situe	Sente	Sauve
Moque	4 → 3	Mitre	Mince	Morue
Pause	4 → 3	Peine	Poche	Panse
Lieue	4 → 3	Lange	Lampe	Ligue
Dames	4 → 3	Doits	Dicos	Dates
Chars	4 → 3	Codes	Colis	Chers
Reste	4 → 3	Ruine	Rafle	Rente
Avant	4 → 3	Admet	Argot	Avent
Potes	4 → 3	Pairs	Panas	Pores
Crocs	2 → 4	Ciels	Cales	Crois
Tract	2 → 4	Tient	Twist	Trait
Celte	2 → 4	Clame	Cadre	Celle
Avale	2 → 4	Arche	Aboie	Avare
Loups	2 → 4	Lents	Laids	Loues
Bulbe	2 → 4	Blase	Barde	Bulle
Parai	2 → 4	Ponti	Publi	Paroi
Proie	2 → 4	Pulpe	Palme	Proue
Tract	2 → 4	Tient	Tuent	Trait
Rende	2 → 4	Riche	Rampe	Renie
Venge	2 → 4	Vulve	Vampe	Venue
Maths	2 → 4	Meurs	Munis	Mates
Plats	2 → 4	Pneus	Peuts	Plans
Porcs	2 → 4	Peins	Palis	Pores
Chaos	2 → 4	Crues	Cries	Chars
Mines	2 → 4	Muscs	Motos	Minus
Tarte	2 → 4	Tique	Toute	Tarie
Remet	2 → 4	Riant	Ragot	Remit
Orale	2 → 4	Ogive	Ozone	Orage
Chefs	2 → 4	Crans	Coups	Chers
Salue	2 → 4	Singe	Score	Salie
Boude	2 → 4	Blase	Berne	Boule
Soirs	2 → 4	Seuls	Sauts	Soies
Plais	2 → 4	Pneus	Pures	Plans
Couve	2 → 4	Clame	Campe	Coule
Diras	2 → 4	Dents	Duels	Dires
Parte	2 → 4	Pulse	Pende	Parue
Finis	2 → 4	Feras	Fards	Fines
Salis	2 → 4	Serfs	Shows	Sales
Tente	2 → 4	Tuile	Tarte	Tenue
Volet	4 → 2	Vivat	Vingt	Valet
Arbre	4 → 2	Azyme	Acide	Ambre
Types	4 → 2	Tubas	Tests	Tapes
Agent	4 → 2	Assit	Ajout	Aient
Fasse	4 → 2	Figue	Fibre	Fusse
Armes	4 → 2	Abois	Avons	Aimes
Mains	4 → 2	Matos	Muses	Moins
Peint	4 → 2	Pavot	Parut	Point
Puise	4 → 2	Phare	Pagne	Prise
Menus	4 → 2	Mugis	Maths	Minus
Pries	4 → 2	Pumas	Ponts	Paies
Divin	4 → 2	Doyen	Dugon	Devin
Ruant	4 → 2	Remit	Robot	Riant
Tendu	4 → 2	Tabou	Tribu	Tondu
Cuves	4 → 2	Comas	Coups	Caves
Tares	4 → 2	Tunis	Thons	Tires
Ravis	4 → 2	Roues	Ruons	Revis
Pendu	4 → 2	Pilou	Panse	Pondu
Tuent	4 → 2	Trait	Tarot	Tient
Mener	4 → 2	Mugir	Major	Miner
Agile	4 → 2	Abuse	Adore	Asile
Moche	4 → 2	Marie	Masse	Miche
Seins	4 → 2	Sumos	Surfs	Soins
Aigle	4 → 2	Anone	Ardue	Angle
Fluet	4 → 2	Fagot	Finit	Fouet
Pales	4 → 2	Punis	Poufs	Piles
Pains	4 → 2	Puces	Ponds	Peins
Fugue	4 → 2	Folie	Fable	Figue
Antre	4 → 2	Avoue	Aboie	Autre
Lames	4 → 2	Logis	Loups	Limes

## Footnotes

1 Flanker effects have also been found using orthographically related words as flankers (Snell, Bertrand, Meeter, & Grainger, 2018; Snell, Vitu, & Grainger, 2017). Furthermore, studies comparing the effects of repeated word flankers with both an unrelated flanker and a no-flanker condition (Snell & Grainger, 2018) have highlighted the contribution of both facilitatory and inhibitory influences of flanking stimuli.

2 It should be noted that 28.05% of the migration flankers and 11.67% of the illusory words did not have an entry in the Lexique database and therefore were not included in these calculations. Pair-wise *t* tests revealed no significant differences between the different migration positions (*p*s > .05).

3 English examples taken from Davis and Bowers (2004).

4 Presentation order (i.e., having seen the target word in the letter migration or control condition first) did not have a significant influence (*b* = −0.07, *SE* = 0.09, *z* = −0.81) and did not interact with the effect of condition (*b* = 0.45, *SE* = 0.25, *z* = 1.77). Furthermore, because some illusory words did appear as targets (*N* = 23), we examined whether seeing the word in advance made a difference in misreporting it as an illusory word. No significant effect was observed (*b* = 22.33, *SE* = 26.69, *z* = 0.83). It should also be noted that the average distance between an illusory word seen before as a target and that illusory word being reported was 87 trials.

5 The same pattern of effects was obtained when including word frequency as a continuous variable in the LME analyses.
